# Central venous catheter use in severe malaria: time to reconsider the World Health Organization guidelines?

**DOI:** 10.1186/1475-2875-10-342

**Published:** 2011-11-14

**Authors:** Josh Hanson, Sophia WK Lam, Sanjib Mohanty, Shamshul Alam, Md Mahtab Uddin Hasan, Sue J Lee, Marcus J Schultz, Prakaykaew Charunwatthana, Sophie Cohen, Ashraf Kabir, Saroj Mishra, Nicholas PJ Day, Nicholas J White, Arjen M Dondorp

**Affiliations:** 1 Department of Medicine, Cairns Base Hospital, Queensland, Australia; 2 Mahidol Oxford Research Unit, Mahidol University, Bangkok, Thailand; 3 Department of Medicine, Ispat General Hospital, Rourkela, India; 4 Department of Intensive Care Medicine, Chittagong Medical College Hospital, Chittagong, Bangladesh; 5 Department of Medicine, Chittagong Medical College Hospital, Chittagong, Bangladesh; 6 Department of Intensive Care Medicine, Academic Medical Center, University of Amsterdam, Amsterdam, The Netherlands; 7 Department of Vascular Medicine, Academic Medical Center, University of Amsterdam, Amsterdam, the Netherlands; 8 Department of Anaesthetics, Cox's Bazar Medical College, Cox's Bazar, Bangladesh; 9 Nuffield Department of Clinical Medicine, University of Oxford, Oxford, UK

## Abstract

**Background:**

To optimize the fluid status of adult patients with severe malaria, World Health Organization (WHO) guidelines recommend the insertion of a central venous catheter (CVC) and a target central venous pressure (CVP) of 0-5 cmH_2_O. However there are few data from clinical trials to support this recommendation.

**Methods:**

Twenty-eight adult Indian and Bangladeshi patients admitted to the intensive care unit with severe *falciparum *malaria were enrolled in the study. All patients had a CVC inserted and had regular CVP measurements recorded. The CVP measurements were compared with markers of disease severity, clinical endpoints and volumetric measures derived from transpulmonary thermodilution.

**Results:**

There was no correlation between the admission CVP and patient outcome (p = 0.67) or disease severity (p = 0.33). There was no correlation between the baseline CVP and the concomitant extravascular lung water (p = 0.62), global end diastolic volume (p = 0.88) or cardiac index (p = 0.44). There was no correlation between the baseline CVP and the likelihood of a patient being fluid responsive (p = 0.37). On the occasions when the CVP was in the WHO target range patients were usually hypovolaemic and often had pulmonary oedema by volumetric measures. Seven of 28 patients suffered a complication of the CVC insertion, although none were fatal.

**Conclusion:**

The WHO recommendation for the routine insertion of a CVC, and the maintenance of a CVP of 0-5 cmH_2_O in adults with severe malaria, should be reconsidered.

## Background

The introduction of artemisinin-based therapy has revolutionized the care of patients with malaria. However, even with intravenous artesunate, the mortality rate of adults with severe malaria in many Asian hospitals remains over 15%; the majority dying during the first 48 hours of their hospitalization [1].

Guidelines for the initial management of patients with sepsis make a series of recommendations based on data collected in multicentre trials [2]; adherence to these guidelines has been shown to improve patient outcomes [3,4]. In contrast, there has been a paucity of clinical trials of patients with malaria and so treatment recommendations for early supportive care are still based largely on expert opinion [5,6].

The management of a patient's fluid status is one of the most fundamental aspects of supportive care. However, clinical assessment of the degree of hypovolaemia is challenging [7] and there is frequently great inter-observer variability [8]. To overcome the limitations of physical examination, critically ill patients will commonly have a catheter inserted into a large central vein to measure the central venous pressure (CVP). CVP is an objective measure of the filling pressure of the heart and has been used in the critical care setting to guide the administration of replacement fluids. In the current Surviving Sepsis Guidelines, fluid resuscitation to achieve a CVP of 8-12 mmHg is one of the key recommendations [2]. Optimizing the fluid status of patients with severe malaria also has appeal as this group has a reduced effective circulating volume [9-11] and lactic acidosis and renal impairment - both exacerbated by reduced tissue perfusion - are important contributors to mortality [12]. Fluid resuscitation has the added advantage of being simple and inexpensive to implement.

Yet in adults with severe malaria, there is also an increase in pulmonary capillary permeability and pulmonary oedema is an important cause of death [13]; hence the old malariologist's adage: "run them dry". This is recognized in the WHO guidelines where experts recommend the use of a central venous catheter (CVC) and a target central venous pressure (CVP) of 0-5 cmH_2_O to address the "very thin dividing line between overhydration, which may produce pulmonary oedema, and underhydration contributing to shock and worsening acidosis and renal impairment" [5].

However, the insertion of a CVC may be associated with mechanical, haemorrhagic and infectious complications [14]. Patients with severe malaria may have thrombocytopaenia and coagulopathy increasing the risk of haemorrhage. The vast majority of patients with severe malaria will have to be managed in a resource-poor environment and in this setting a CVC is relatively costly. Furthermore there has recently been a move away from reliance on pressure-based measures of preload given the limited correlation between such measures and true volume status [15,16].

Thus, there is a concern that an expensive intervention is being recommended which could increase risks for the patient without providing useful guidance for the clinician. As part of a study examining fluid resuscitation of adults with severe malaria we aimed to determine the utility of CVP in the management of this group of patients.

## Methods

### Study

The primary study, of which this analysis is a sub-study, examined the response of adult patients with severe *Plasmodium falciparum *malaria to fluid loading. Ethical approval for the study was obtained from the Bangladeshi Medical Research Council, the Institutional Ethical Board of Ispat General Hospital, and the Oxford Tropical Medicine Research Ethical Committee. There were regular scheduled reviews of the study by independent local safety committees. Patients were enrolled only after written informed consent was obtained from an accompanying relative via a local translator.

### Patients

Patients were recruited at Chittagong Medical College Hospital in Bangladesh and Ispat General Hospital in Rourkela, India in 2008. At both sites malaria is endemic with seasonal peaks in transmission. Patients were defined as having malaria if asexual forms of *P. falciparum *were present on blood film or, if expert microscopy was not immediately available, an immunochromatographic test (Paracheck Pf, Orchid Biomedical Systems, India) was positive. In these cases *falciparum *malaria was later confirmed by microscopic examination of a simultaneously collected slide. As invasive haemodynamic monitoring was being employed, only the sickest patients were enrolled. The prospectively defined severity criteria were: a peripheral venous base deficit of > 6 mmol/L, a blood urea nitrogen > 60 mg/dL (21.4 mmol/L) or pulmonary oedema (defined as oxygen saturation < 90% on room air with bi-basal crepitations on respiratory examination). These inclusion criteria were a modification of the WHO criteria for severe malaria and reflected the potential for iatrogenic harm from insertion of the CVC and arterial catheter. Patients were excluded if they were < 16 years old or if they had already received artesunate for > 24 hours prior to enrolment.

### Treatment protocol

All patients were admitted to the intensive care unit (ICU). A detailed history and physical examination was performed on all patients who were treated with intravenous artesunate and standard supportive care as per current treatment guidelines [5]. Jugular venous pressure (JVP) was determined by inspecting the internal jugular vein (IJV) or the external jugular vein if the IJV was not seen. The JVP was classified as not visible, just visible, normal, or elevated. A 7 French 20 centimetre triple lumen central venous catheter (CVC) (Arrow, North Carolina, USA) was placed by an clinician experienced in insertion of central venous catheters using the Seldinger technique in either the subclavian vein (SCV) or the IJV; the SCV was preferred unless the patient had respiratory compromise. A chest x-ray was performed to confirm the correct positioning of the CVC and exclude complications of its insertion. CVP was then recorded using a manometer connected to the patient and a flask of saline via a three-way tap, the level of the right atrium was designated as the zero point. Nurses in both ICUs were unfamiliar with CVCs and so received training during the course of the study. The CVC insertion site was dressed daily with 2% chlorhexidine and non-occlusive sterile gauze. Further haemodynamic measurements were made using transpulmonary thermodilution and arterial pulse contour analysis (PiCCO plus^®^, Pulsion, Germany) using the CVC and a 5 French thermistor-tipped arterial catheter (Pulsiocath, Pulsion, Germany) inserted into the femoral artery. The physiological basis for measurement of haemodynamic variables using the PiCCO system has been published in detail elsewhere [17,18]. Briefly, chilled saline (< 8° Celsius) was injected into the superior vena cava via the CVC and the thermistor at the tip of the femoral artery catheter measured the downstream temperature change. Cardiac output was calculated by analysis of the thermodilution curve using a modified Stewart-Hamilton algorithm. All volumetric parameters were obtained by advanced analysis of the thermodilution curve. These parameters included global end diastolic volume (GEDI) - a measure of volume status - and extra vascular lung water (EVLW). The patient's height was used to calculate the ideal body weight for the calculation of indexed values.

Patients were resuscitated with isotonic saline using published PiCCO measurement based, CVP independent, resuscitation algorithms [19,20]. Blood was transfused if the haemoglobin fell below 5 g/dL. Repeat Respiratory support, renal replacement therapy (RRT) and inotropic support were initiated based on the judgement of the clinicians. Disease severity was determined using the "CAM" score [12].

### Laboratory

Thick and thin blood films for parasite quantification, a full blood count and plasma biochemistry were collected on admission. On admission and during the study measures of biochemistry and haemoglobin were determined by a handheld automated analyser (iStat, Abbott Laboratories, New Jersey, USA). Sequential blood films to confirm parasitological response were collected. Blood cultures were collected in patients in whom concomitant bacterial infection was felt to be possible. Other investigations were initiated according to the clinical judgement of the attending staff.

### Statistics

Data were collected and entered into a de-identified database and analysed using statistical software (Version 10, StataCorp). Correlation coefficients were determined using Spearman's method. Differences between groups were analysed using the Kruskal-Wallis and Chi-squared tests.

## Results

### Patient characteristics

Twenty-eight patients were included in this study, five (17.8%) of whom died. Their baseline characteristics are recorded in Table 1. All the patients were felt to have their clinical presentation explained solely by severe *falciparum *malaria. In the course of their admissions, 11 received respiratory assistance (eight endotracheal intubation and mechanical ventilation, three continuous positive airways pressure ventilation by facemask) and six received RRT (three peritoneal dialysis, three haemodialysis). Seven patients received inotropic support (dopamine), although in five of these this was in the pre-terminal setting.

**Table 1:** Baseline characteristics of the patients

Variable	Median value	Range
Age	36 years	(17-66)

Sex	19/28 (68%) male	-

Heart rate	102 beats per minute	78-126

Mean arterial pressure	84 mm Hg	58-110

Glasgow Coma Score	13	4-15

Oxygen saturation	98 %	86-100

Haemoglobin	10.4 g/dL	5.1-14.3

White cell count	8.0 × 10^9^/L	3-21.4

Platelets	26 × 10^9^/L	8-244

Parasite count	15229 parasites/μl	38-2411897

Sodium	134 mmol/L	120-150

Potassium	3.9 mmol/L	2.7-5.1

Blood urea nitrogen	15 mmol/L	2-50*

Creatinine	172 μmol/L	70-862

Bicarbonate	16.9 mmol/L	6.6-22

Lactate	3.1 mmol/L	0.46-12.8

Base deficit	-9 mmol/L	-4 - -23

Central Venous Pressure	5.25 cm H_2_O	-7 to 13

Global End Diastolic Index	481 ml/m^2^	346-675

Extra Vascular Lung Water	8 ml/Kg	5-14

Cardiac Index	3.1 L/min/m^2^	2.27-5.24

Oxygen delivery (DO_2_)	420 ml/min/m^2^	253-603

Central venous O_2 _saturation (SCVO_2_)	64%	48-85

CAM score	3	1-4

Histidine Rich Protein 2 (HRP2)	6.1 μg/mL	1.2-34.6

* The limit of detection using the IStat system.

### CVP and GEDI

The median (range) CVP on admission to the study was 5 (-7 to 13) cmH_2_O. There was neither an association between admission CVP and disease severity (p = 0.33), nor between admission CVP and patient outcome (5 (-7 to 13) cmH_2_O in survivors versus 6 (-3 to 9) cmH_2_O in those who died, p = 0.67). There was no association between admission CVP and serum creatinine (p = 0.57), blood urea nitrogen (p = 0.40), venous lactate (p = 0.34) or base deficit (p = 0.58). Admission CVP was higher in patients requiring respiratory assistance during admission (7 (-3 to 9) cmH_2_O versus 2 (-7 to 13) cmH_2_O), but this did not reach statistical significance (p = 0.06) and there was a large overlap between patients who did and did not require this supportive treatment. Admission CVP in patients was also higher in patients requiring dialysis later during admission (7 (4 to 9) cmH_2_O versus 3 (-7 to 13) cmH_2_O). Once again this did not reach statistical significance (p = 0.08) and there was a large overlap between the two groups.

**Figure 1:**
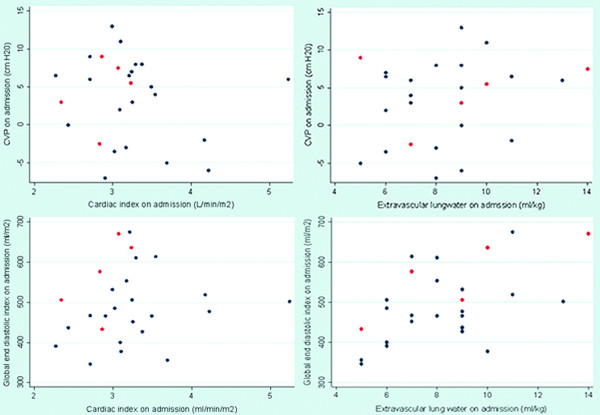
**Association between haemodynamic variables at baseline**. a) CVP versus cardiac index on admission, p = 0.44 r_s _= -0.16. Blue dots survivors, red dots: deaths. b) CVP versus extravascular lung water on admission, p = 0.62 r_s _= 0.1 Blue dots survivors, red dots: deaths. c) Global end diastolic volume versus cardiac index on admission, p = 0.35 r_s _= 0.1. Blue dots survivors, red dots: deaths. d) Global end diastolic volume versus extravascular lung water on admission, p = 0.01 r_s _= 0.5. Blue dots survivors, red dots: deaths.

All patients were hypovolaemic with a median (range) GEDI on admission of 481 (346 to 675) ml/m^2 ^(normal > 680 ml/m^2^). There was a correlation between admission GEDI and EVLW (p = 0.01), but not between admission GEDI and CI (p = 0.35). There was no relationship between admission GEDI and mortality (median (range) 467 (346 to 675) in survivors versus 577 (433 to 671) in fatal cases; p = 0.09). There was no correlation between admission GEDI and the base deficit (p = 0.29) or CAM score (p = 0.30). There was no relationship between admission GEDI and serum creatinine or blood urea (p = 0.98 for both). There was also no relationship between admission GEDI and the later need for RRT (p = 0.36) or respiratory assistance (p = 0.11). There was correlation between admission GEDI and serum lactate (p = 0.05), but lower serum lactate levels were seen in patients with a lower GEDI. Notably, there was no correlation on admission between the admission CVP and the GEDI (p = 0.88), the CI (p = 0.44) or the EVLW (p = 0.62) (Figure 1).

### Volume responsiveness

As the GEDI was used to guide resuscitation, the correlation between admission GEDI and volume of fluid administered was expected (r_s _= -0.55, p = 0.004). There was no relationship between the CVP on admission and the volume of fluid required to resuscitate the patient (r_s _= -0.14, p = 0.47).

**Figure 2:**
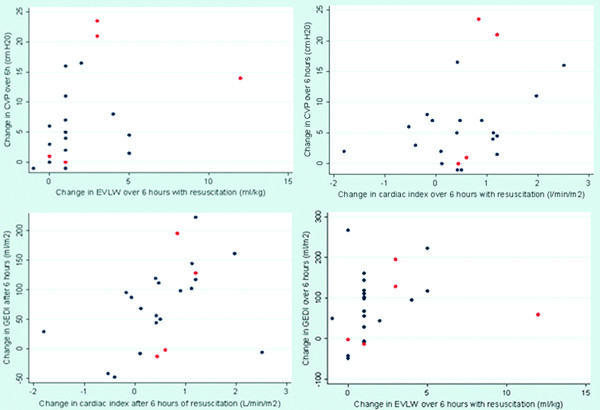
**Association between change in haemodynamic variables with fluid loading**. a) ΔCVP versus Δ cardiac index after 6 hours of fluid resuscitation, p = 0.24 r_s _= 0.26. Blue dots survivors, red dots: deaths. b) ΔCVP versus Δ extravascular lung water after 6 hours of fluid resuscitation, p = 0.005 r_s _= 0.54. Blue dots survivors, red dots: deaths. c) ΔGEDI versus Δ cardiac index water after 6 hours of fluid resuscitation, p = 0.004 r_s _= 0.58. Blue dots survivors, red dots: deaths. d) ΔGEDI versus Δ extravascular lung water after 6 hours of fluid resuscitation, p = 0.056 r_s _= 0.39. Blue dots survivors, red dots: deaths.

The change in GEDI (ΔGEDI) with six hours of fluid loading was correlated with the change in CI (ΔCI) (p = 0.004, r_s _= 0.58), but there was no correlation between change in CVP (ΔCVP) and ΔCI (p = 0.24, r_s _= 0.26). Of the 28 patients, 12 were fluid responsive (> 15% increase in CI over 6 h of fluid loading). Neither the baseline CVP (p = 0.37) or baseline GEDI (p = 0.07) predicted whether the patient would be fluid responsive. There was borderline correlation between ΔGEDI and the change in EVLW (ΔEVLW, p = 0.06, r_s _= 0.39). There was a correlation between ΔCVP and ΔEVLW (p = 0.01, r_s _= 0.54), however there was a large overlap in values: a change in EVLW of 1 ml/kg was associated with a change in CVP of anywhere from -1 to 16 cm H_2_O (Figure 2).

### Correlations with the WHO target range

The patients had a median (interquartile range (IQR)) of 15 (11 to 18) CVP measurements taken over 96 hours. A median of 17% (6 to 39) of CVP measurements for each patient fell in the WHO recommended range of 0 to 5 cmH_2_O. Twenty-three (78%) of the patients had at least one CVP measurement in the range of 0 to 5 cmH_2_O. Twenty of these 23 patients had either hypovolaemia (GEDI < 680), pulmonary oedema (EVLW > 10) or both, at all timepoints when their CVP readings were in this target range. The remaining three patients had PiCCO-defined hypovolaemia or pulmonary oedema at 80%, 80% and 45% of timepoints when their CVP was in the target range (table 2).

**Table 2:** Presence of hypovolaemia or pulmonary oedema whilst CVP in the WHO target range of 0-5 cm H_2_O.

Patient number	Times CVP in target range° n	No pulmonary oedema*, no hypovolaemia^ n (%)	Pulmonary oedema*, no hypovolaemia^ n (%)	Pulmonary oedema*, hypovolaemia^ n (%)	No pulmonary oedema*, hypovolaemia^ n (%)
CN10	15	0	2 (10)	7 (50)	6 (40)

CN14	1	0	0	0	1 (100)

CN15	7	0	0	1(14)	6 (86)

CN18	12	0	0	0	12 (100)

CN20	11	6 (55)	4 (36)	0	1 (9)

CN24	8	0	0	8(100)^¶^	0

CN25	1	0	0	0	1 (100)

CN29	1	0	0	0	1 (100)

CN30	5	0	0	0	5 (100)

RN01	2	0	0	0	2 (100)

RN03	1	0	0	0	1 (100)

RN06	6	0	0	0	6 (100)

RN07	1	0	0	0	1 (100)

RN08	2	0	0	0	2 (100)

RN09	1	0	0	1(100)	0

RN10	2	0	0	0	2 (100)

RN11	5	1 (20)	0	0	4 (80)

RN12	3	0	0	0	3 (100)

RN13	5	0	0	0	5 (100)

RN14	7	0	1 (14)	1 (14)	5 (72)

RN17	1	0	0	1(100)	0

RN18	5	1 (20)	3 (60)	0	1 (20)

RN19	2	0	0	0	2 (100)

°WHO target CVP: 0-5 cm H_2_O

*Pulmonary oedema:EVLW > 10 ml/kg

^Hypovolaemia: GEDI < 680 ml/m^2^

^¶ ^This patient had aspiration pneumonia, confounding this measurement.

### Correlation with JVP

The jugular venous pressure and central venous pressure were measured simultaneously on 368 occasions. There was a strong correlation between the CVP and JVP (r_s _= 0.47, p < 0.001) however there was no correlation between the JVP and the GEDI (r_s _= 0.07, p = 0.16), CI (r_s _= 0.02, p = 0.69) or EVLW (r_s _= -0.05, p = 0.32).

### Complications

There were significant complications of CVC insertion in 7 (25%) patients, including aspiration of stomach contents during the procedure (n = 2) and blood stream infection (n = 3). Persistent oozing of blood around the exit site following insertion necessitated the removal of the CVC in two cases. There was no pneumothorax. Although there were no fatal complications, all required an escalation of therapy.

## Discussion

The data collected in this study show that in adult patients with severe malaria CVP is not a reliable predictor of volume status, nor is it a clinically useful measure of the likelihood of developing pulmonary oedema, and therefore cannot be used as a guide for fluid therapy. The data collected in this study do not support the WHO recommendation that a CVP of 0-5 cm H_2_O should be used as a resuscitation target; the vast majority of all patients with a CVP recorded in the target range had concurrent hypovolaemia, pulmonary oedema or both while their CVP was in the target range. There was no correlation between the baseline CVP and the likelihood of the cardiac index (CI) being volume responsive, nor between the change in CVP (ΔCVP) and change in CI with fluid loading. While ΔCVP was correlated with the change in lung water with fluid loading there was such a large overlap in values that CVP is rendered clinically useless as a predictor in this situation. There was no correlation between CVP and renal function.

There is no literature on the use of CVP as a resuscitation target in patients with severe malaria, however pulmonary oedema has been shown not to be correlated with either elevated CVP or pulmonary artery occlusion pressure in previous studies of this group of patients [21,22]. In the only large study to examine their relationship, measurements of CVP were actually lower in patients with pulmonary oedema than those in patients without this complication [23].

In recent years, there has been a reduction in the emphasis on pressure-based measures as endpoints for resuscitation in critically ill patients [24-27]. CVP is not a good predictor of fluid responsiveness in either healthy volunteers [24] or hypovolaemic critically ill patients [26], and current opinion in well equipped intensive care units is that CVP measurement alone should not be used to define the state of ventricular filling (preload) or the potential of patients to respond to a fluid challenge [15,16].

Adherence to the bundle of care recommended in the Surviving Sepsis Guidelines has been shown to improve the outcomes of patients suffering from severe sepsis. Fluid resuscitation to a CVP of ≥ 8 mmHg in patients with persistent hypotension is one of the key tenets of these guidelines. However, when different components of the bundle of care are independently assessed investigators have shown that there was no association between achieving this CVP target and outcome [28,29]. This has led some authors to question the appropriateness of a CVP target in guidelines [30].

Insertion of CVCs is not without risks: mechanical complications occur in 5-19% of patients, infectious complications occur in 5-26%, and thrombotic complications occur in 2-26% [14]. Patients with severe malaria have thrombocytopenia and sometimes coagulopathy increasing the risk of haemorrhage. CVCs are relatively expensive, require medical and nursing expertise to insert and maintain and supporting radiology and microbiology services; all of which will often be lacking in the resource-poor settings where most patients with severe malaria are managed. In our series 25% of the patients had a significant complication of CVC insertion - a relatively high figure, but indicative of the resource-poor setting in which the patients were managed.

Central venous access for inotrope therapy is needed in only a minority of patients, since shock is a rare complication in severe malaria [1]. Also, parenteral feeding in patients with coma is seldom indicated, since coma recovery is generally within 72 hours [1].

In our study the JVP was strongly correlated with CVP, a relationship that has been noted in other studies [8,31] although the fact that in this study the JVP and CVP were measured by the same examiners concurrently potentially confounds this observation. However, there was similarly little correlation between the JVP and measures of volume status, pulmonary oedema or cardiac index or volume responsiveness. Furthermore JVP is a notoriously challenging sign to interpret, considerable variation exists in expertise in the assessment of JVP, and some studies have shown poor reliability of such assessments in critically ill patients [32,33].

Ultimately, the goal of fluid therapy with the optimization of blood volume is to improve cardiac output and hence oxygen delivery to tissues. However the central pathophysiological mechanism of severe falciparum malaria is the disturbance of microcirculatory blood flow. The adherence of parasitized red blood cells to the endothelium - compounded by auto-agglutination, rosetting and reduced deformability of the red cells - leads to decreased tissue perfusion and the lactic acidosis which has repeatedly been shown to be the strongest predictor of mortality. Microvascular measures like the proportion of obstructed capillaries in rectal mucosa or estimates of the sequestered parasite biomass, have been shown to be strongly correlated with lactic acidosis and outcome in patients with severe malaria [34,35]. Fluid loading to systemic haemodynamic targets may do little to overcome this microcirculatory obstruction whereas pulmonary oedema associated with fluid loading in malaria is common and has a mortality rate in adults in resource-poor settings of up to 80%[13]. Recent data collected in African children with severe malaria have demonstrated increased mortality in children treated with aggressive fluid loading [36].

PiCCO derived volume based measures were more reliable as predictors of fluid responsiveness in this group of patients, as has been reported previously [37-40] However, the required expertise and resources limit its feasibility for deployment in the field. Moreover management of critically ill patients using PiCCO based measures has not yet been shown to improve outcome when compared to pressure based systems [41].

There are some important limits to the study. Only a small number of patients were enrolled and PiCCO derived measures were used as our gold standard measure of CI, GEDI and EVLW. The PiCCO system has not been validated in patients with severe malaria; it is theoretically possible that the microcirculatory derangement seen in malaria might affect the Stewart-Hamilton algorithm. The correlation seen between PiCCO measures may be argued to be the result of mathematical coupling although previous research has suggested that this is not the case [42]. The study's findings do not preclude the possible utility of central venous access in some patients with severe malaria: central venous access would have value in patients requiring inotropic support or in whom peripheral access is not possible.

In summary, the data collected in this study show that invasive CVP measurement in adult patients with severe malaria provides little clinically useful information, while carrying appreciable morbidity and significant costs relevant to the resource-poor setting in which most patients are managed. In light of these data the WHO recommendations for the routine use of central venous lines - and a target CVP of 0-5 cm H_2_O - in patients with severe malaria should be reconsidered.

## Competing interests

The authors declare that they have no competing interests.

## Authors' contributions

JH devised and performed the study, performed the statistical analysis and was the primary author of the manuscript. SWKL and SC performed the study and collected data. SM, SA, MMUH, AK, SM supervised the study. PC assisted with the logistical preparation and implementation of the project. SJL assisted with the statistical analysis. MJS, NPJD and NJW reviewed and revised the manuscript. AMD was the primary supervisor and revised the manuscript. All authors reviewed the manuscript, and read and approved the final version.
